# A Process-Based Model for Bioturbation-Induced Mixing

**DOI:** 10.1038/s41598-017-14705-1

**Published:** 2017-10-27

**Authors:** Tomás Aquino, Kevin R. Roche, Antoine Aubeneau, Aaron I. Packman, Diogo Bolster

**Affiliations:** 1 Spanish National Research Council (IDAEA – CSIC), 08034 Barcelona, Spain; 2 Department of Civil and Environmental Engineering and Earth Sciences, University of Notre Dame, 46556 Indiana, USA; 3 Department of Civil and Environmental Engineering, Northwestern University, 60208 IL, USA; 4 Lyles School of Civil Engineering, Purdue University, 47907 Indiana, USA

## Abstract

Bioturbation refers to the transport processes carried out by living organisms and their physical effects on soils and sediments. It is widely recognized as an important mixing mechanism, particularly at the sediment-water interface in many natural systems. In order to quantify its impact on mixing, we propose a process-based model based on simple assumptions about organism burrowing behavior. Specifically, we consider burrowing events to be stochastic but memoryless, leading to exponential inter-burrow waiting times and depths. We then explore the impact of two different transport mechanisms on the vertical concentration distributions predicted by the model for a conservative (inert) tracer. We compare the results of our model to experimental data from a recent laboratory study of bioturbation by the freshwater oligochaete worm *Lumbriculus variegatus*, and find good quantitative agreement.

## Introduction

Bioturbation refers to the transport processes carried out by living organisms which affect soils and sediments^1^. Organisms actively rework soils and sediments by creating burrows, feeding, and seeking refuge. This physical reworking provides an important control on interfacial mixing and structure in many terrestrial and aquatic ecosystems, which can profoundly impact the chemistry and ecology of sediments^2^. In particular, it represents the dominant mode of sediment transport in many freshwater, coastal, and marine systems^1,3^. In the absence of high-shear events such as storms, bioturbation is often the main driver of vertical mixing of solutes and particulates at and below the sediment-water interface, where chemical conditions may vary substantially^3,4^. Thus, modeling the fate and transport of deposited or adsorbed substances such as organic matter, contaminants, and nutrients in the subsurface often requires adequate understanding and parameterization of bioturbation mechanisms.

Early models of bioturbation assumed transport to be described by an effective vertical velocity and Gaussian fluctuations, leading to a one-dimensional advection–dispersion equation^2,5–8^. This model, often termed biodiffusion, was later extended to account for experimentally observed non-Fickian characteristics, for example by including a nonlocal transport term representing fast transport between the surface and deeper sediment regions^2,9–11^. More recently, continuous time random walk models were employed to better capture experimental observations and reduce the severity of asymptotic timescale assumptions associated with advection–dispersion models^12,13^. The relationship of these models to the more standard biodiffusion model is also well understood^14,15^. However, predictive application of bioturbation models to varying real-world scenarios requires a parameterization in terms of physical properties that may be measured or estimated *a priori*, a challenge that remains largely an open problem. Model validation is also currently limited by the lack of direct observations of organism activity and resulting sediment transport, which are challenging to observe directly^3^.

The aim of the present study is to develop a process-based model for vertical bioturbation-induced mixing. Such a model is meant to help examine the consequences of simple, easily interpretable mechanisms, and to provide a basis for parameterization based on physical reasoning and appropriately designed experimental studies. In order to assess the applicability of our model, we compare its predictions for vertical concentration profiles of an inert tracer to recent observations from a laboratory study of bioturbation by *Lumbriculus variegatus* ^16^, an oligochaete worm commonly found in freshwater settings^17–19^.

## Model

In this section, we describe the assumptions underlying our model, discuss their implications, and present some analytical considerations in order to obtain time-dependent tracer distributions. We also present a standard one-dimensional advection–dispersion model with constant coefficients for comparison.

Our goal is to develop a parsimonious model based on minimal assumptions about organism behavior, while retaining stochastic variability at the level of burrowing activity. Our mixing model has two conceptual components: a burrowing description, and a transport description linking burrowing activity to tracer displacement. The model’s assumptions are:

1. Each organism initiates a burrowing event with a fixed probability per unit time *γ*. The burrowing location is chosen at random with all locations equally probable.
2. During a burrowing event, the burrow is excavated at a fixed downward velocity. The termination of burrowing is independent of previous events and occurs with a fixed probability per unit time; the organism subsequently returns to the interface. The duration of burrow construction is assumed small compared to the time scale over which new burrowing events occur, and is neglected.
3. During a burrowing event, tracer transport is carried out according to one of the following rules:

- (a) Tracer encountered is spread uniformly across the burrowing depth.
- (b) Tracer encountered is transported to the bottom of the burrow.

Points 1. and 2. comprise the burrowing description. Point 3. introduces two variants of the transport description; these will henceforth be referred to as (a) and (b).

### Assumption 1

describes memoryless burrowing. It implies that the total number of burrowing events by time *t* is Poisson-distributed with mean *γt*, or equivalently that the waiting time between burrowing events is exponentially distributed with mean waiting time 1/*γ*. This follows because the exponential waiting time is the only memoryless stochastic process, such that events are characterized by a fixed probability of occurrence per unit time. The corresponding number of events in a given time window is Poisson-distributed^20^. Discretizing the sediment-water interface domain of interest into *M* bins with an area on the order of the typical horizontal cross-section of a burrow, a simple combinatorial argument shows that the probability of a given burrowing event occurring at a specific location (i.e. horizontal position on the interface) is Binomial-distributed with a probability of success equal to 1/*M*. Assuming there are *N* organisms present, this implies that the number of burrowing events by time *t* at a specific location is again Poisson-distributed with mean *βt*, where *β* = *γN*/*M* is the mean burrowing rate rescaled by a dimensionless organism number density representing the number of organisms per potential burrowing location. Equivalently, the waiting time between burrowing events at a given location, due to any organism, is exponentially distributed with mean 1/*β*.

### Assumption 2

states that organisms burrow without memory and at a uniform speed during a burrowing event. The short duration of burrow construction compared to the overall time scale over which new burrowing events occur is qualitatively supported in the context of our case study by the time-lapse videos collected during the original experiment^16^. The assumption of immediate return to the interface is adequate for highly motile, aerobic organisms (which is the case for *L*. *variegatus*). Lack of memory and one-dimensionality imply that the depth attained by a single burrowing event is exponentially distributed with some mean 1/*α*.

**Figure 1:**
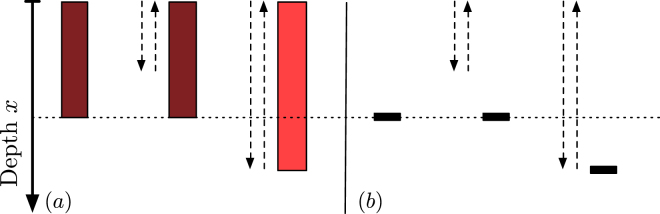
Illustration of transport rules (**a**,**b**). In both cases, a burrowing event (dashed arrows) at a given location has no effect on the tracer distribution if it does not attain a depth exceeding the maximum previously attained depth (horizontal dashed line). For rule (**a**), a burrowing event exceeding the current depth at a given location homogenizes the tracer over the attained depth. For rule (**b**), a burrowing event exceeding the current depth at a given location displaces the tracer to the bottom of the burrow.

The transport descriptions (a) and (b) in assumption 3. may be seen as end-members with regard to tracer mixing efficiency during a burrowing event. These descriptions allow us to explore the impact of different assumptions regarding tracer transport in the framework of our burrowing model. Both assumptions have an important consequence for the dynamics that simplifies the analysis of vertical concentration distributions. To see this, consider a single burrowing site. At a given time, a certain maximum burrowing depth *x*_*m*_ will have been attained. In model (a), tracer mass is uniformly distributed for all depths *x* < *x*_*m*_, and in model (b), all mass is at depth *x*_*m*_. According to either transport rule, subsequent burrowing events that do not exceed depth *x*_*m*_ have no impact on the tracer distribution (see Fig. 1).

Our model is similar in spirit to a continuous time random walk approach, in that we consider stochastic inter-burrowing times and burrowing depths^13,15^. However, we emphasize the development of a mechanistic model of burrow construction in combination with a tracer transport model. Based on the assumptions discussed above, we show in the next section how vertical concentration profiles may be obtained in a straightforward manner and in terms of parameters with a clear physical meaning.

## Tracer Distributions

We are now interested in computing vertical (cross-section-averaged over the horizontal directions, that is, at each depth over all potential burrowing locations) tracer concentration profiles. We assume the initial tracer concentration to be described by a Dirac delta pulse located at the sediment-water interface. This choice is appropriate to model the experimental setup discussed in the next section; the model may be easily generalized to describe other initial conditions (by convolving the profiles obtained here with the initial distribution). We normalize concentrations to unit integral over depth at each time, and consider depths relative to the interface.

As explained above, the maximum depth at a given burrowing location and at a given time plays a central role. The exponential character of the waiting times between burrowing events and the exponential distribution of depths for each burrowing event imply that the probability density of the maximum depth at a given location is double exponential^21^:

1 $${p}_{max}(x,t)={e}^{-\beta t}\delta (x)+\alpha \beta t\,\exp \,[-(\alpha x+\beta t{e}^{-\alpha x})],$$

where the term proportional to the Dirac delta *δ*(*x*) accounts for the probability of no burrowing events having yet occurred at that location.

For model (a), at a given depth *x*, only sites with current maximum depth *x*_*m*_ > *x* contribute a nonzero concentration, of magnitude 1/*x*_*m*_. For each value of *x*_*m*_ > *x*, this happens with probability *p*_*max*_(*x*_*m*_, *t*). The tracer concentration as a function of depth *x* is then:

2 $${c}_{a}(x,t)={\int }_{x}^{\infty }\,\frac{{p}_{max}({x}_{m},t)}{{x}_{m}}\,{\rm{d}}{x}_{m}.$$

This integral was evaluated numerically.

For model (b), the tracer concentration at a given location is a Dirac delta pulse located at the current maximum depth, so that:

3 $${c}_{b}(x,t)={p}_{max}(x,t\mathrm{).}$$

We are also interested in computing the mean *μ*(*t*) and variance *σ* ^2^(*t*) of these profiles as a function of time *t*. For model (a), we evaluate these two metrics by appropriate numerical integration. For model (b), closed-form solutions are available in terms of special functions:

4 $$\begin{array}{ccc}{\mu }_{b}(t) & = & \frac{{\gamma }_{E}+{\rm{\Gamma }}(0,\,\beta t)+\,{\rm{l}}{\rm{o}}{\rm{g}}(\beta t)}{\alpha },\\ {\sigma }_{b}^{2}(t) & = & \frac{2\beta t\,{}_{3}{F}_{3}(1,1,1;2,2,2;-\beta t)}{{\alpha }^{2}}-{\mu }_{b}^{2}(t),\end{array}$$

where *γ*_*E*_ is Euler’s constant, Γ is the incomplete Gamma function, and _3_ *F*_3_ is a hypergeometric function.

For comparison purposes in the next section, the advection–dispersion (biodiffusion) model, assuming constant velocity *v* and constant dispersion *D* for all times and depths, and considering a reflecting boundary at the sediment-water interface, leads to:

5 $$\begin{array}{rcl}{C}_{ADE}(x,t) & = & \frac{1}{\sqrt{4\pi Dt}}\,({e}^{-\frac{{(x-vt)}^{2}}{4Dt}}+{e}^{-\frac{{(x+vt)}^{2}}{4Dt}}),\\ \,\,\,{\mu }_{ADE}(t) & = & vt\,[{\rm{erf}}\,(\frac{1}{2}\sqrt{\frac{{v}^{2}t}{D}})+2\sqrt{\frac{D}{\pi {v}^{2}t}}{e}^{-\frac{{v}^{2}t}{4D}}],\\ \,\,\,{\sigma }_{ADE}^{2}(t) & = & 2Dt+{v}^{2}{t}^{2}-{\mu }_{ADE}^{2}(t\mathrm{).}\end{array}$$

## Results

We now provide comparisons of our model’s predictions for vertical tracer concentration distributions to experimental measurements from a recent study of bioturbation by *L*. *variegatus* ^16^. Specifically, we consider measurements of tracer concentration profiles in a vertically oriented planar cross-section, averaged horizontally at each depth to produce one-dimensional vertical profiles. An inert, fluorescently-labeled particulate tracer was initially injected approximately homogeneously at the sediment-water interface, and a known mass of worms was introduced at time *t* = 0. Profiles were measured every 90 minutes for 15.1 days. We refer the reader to the original study for a detailed description of experimental conditions and measurement techniques.

**Figure 2:**
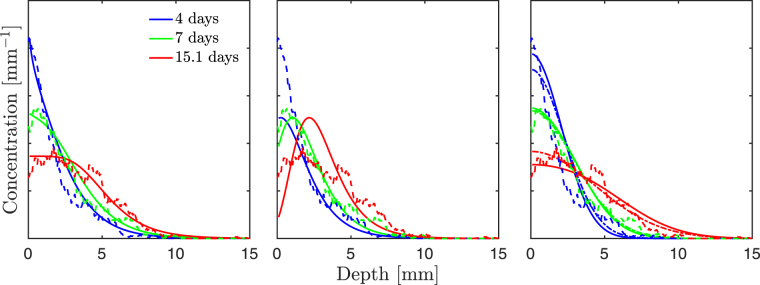
Normalized tracer concentration profiles. Dashed lines correspond to experimental data, and solid lines to model results. The dash-dotted lines in the rightmost plot refer to the constrained ADE model, with *v* = 0. From left to right: model (**a**) (*α* = 5.0 · 10^−1^ mm^−1^, *γ* = 3.1 · 10^3^ day^−1^, *r* ^2^ = 0.95); model (**b**) (*α* = 6.9 · 10^−1^ mm^−1^, *γ* = 9.1 · 10^2^ day^−1^, *r* ^2^ = 0.8); ADE (solid line: *D* = 4.4 · 10^−1^ mm^2^/day^−1^, *v* = 3.0 · 10^−1^ mm/day^−1^, *r* ^2^ = 0.93; dash-dotted line: *D* = 6.3 · 10^−1^ mm^2^ day^−1^, *r* ^2^ = 0.92).

**Figure 3:**
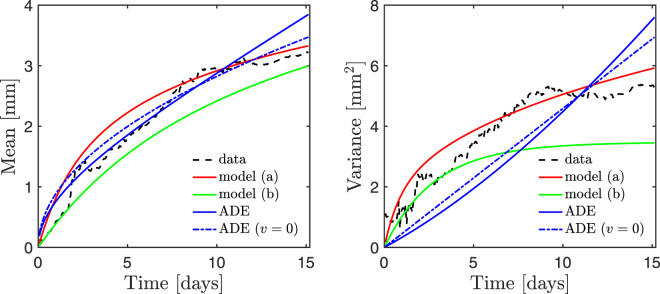
Experimental and model normalized tracer concentration statistics.

In Fig. 2 we show experimental and modeled tracer concentrations as a function of depth at three different times. In Fig. 3 we present the mean and variance of the concentration profiles as functions of time. Parameters *α* and *γ* for models (a) and (b) were obtained by least-square-fitting the experimental profiles. We based the fits on 50 temporally equispaced profiles from days 1.25 through the duration of the experiment, since data at very early times was substantially noisier (likely due to insufficient statistics and/or noise in the initial injection). The number of worms was taken as *N* = 63, in accordance with the original study. The horizontal dimensions of the tank used in the experiment were 10 cm × 10 cm, and the horizontal pixel width of the processed images is 230 *μ*m, the characteristic burrow width, which is approximately the characteristic linear dimension of a worm’s cross-section. The number of bins *M* = 1.9 · 10^5^ was obtained by dividing the total cross-sectional area of the experimental tank by the pixel width squared. Since Dirac delta pulses are mathematical abstractions, we accounted for the small but finite width of the experimental initial condition by considering the origin of our coordinate system to coincide approximately with the lower edge of the initial injection (about 0.3 mm, the halfwidth of the initial condition, below the experimentally determined sediment-water interface).

The parameters (*α* and *γ*, and *D* and *v*) and coefficients of determination (*r* ^2^) resulting from the fitting procedures are given in Fig. 2. We obtained average depths 1/*α* ~ 2 mm and single-worm burrowing rates *γ* ~ 10^3^/day for models (a) and (b). We note that the average depth is small compared to the typical worm length, which is on the order of a centimeter. However, worms were often observed burrowing only partially, with the majority of their length remaining above the sediment-water interface. We recall also that the vertical burrowing assumption is an idealization, and that burrows observed in the experiment were not fully vertical; thus, the average depth parameter may be thought of as an effective vertical depth, which is smaller than the full burrow length. The high values of the burrowing rate mean that models (a) and (b) predict high organism activity, but the low values of *α* mean that burrows are often very shallow, which again is qualitatively consistent with observations. Note, however, that we have reported the single-worm burrowing rate *γ* rather than the single-location burrowing rate *β* in order to provide a description in terms of organism traits. Since the tracer dynamics in models (a) and (b) are determined by *β*, it is important to note that the value of *γ* for a given *β* is proportional to the number of potential burrowing sites *M*, and thus inversely proportional to the square of the characteristic linear dimension of a burrow cross-section. This high sensitivity to our estimate may have led to an overprediction, and further experiments focusing on the single-organism burrowing rate at the sediment-water interface may yield better estimates. For the ADE model, we first optimized the full two-parameter model in terms of *D* and *v*. However, advection is typically considered to represent movement of the sediment-water interface (although mechanisms leading to non-zero advection but no net interface drift have also been proposed^11^). In our model, this effect is absent, given that we consider depths relative to the interface. Thus, we also optimized the model under the constraint *v* = 0. While the unconstrained model performed slightly better according to our fitting procedure, the results are qualitatively similar. We also investigated another two-parameter dispersion model, with a depth-dependent, exponentially decaying dispersion profile *D*(*x*) = *D*_0_ *e* ^−*ρx*^. Surprisingly, we found that this additional structure did not lead to better results: in fact, the best fits were obtained for *ρ* = 0, in which case this model is identical to the standard dispersion equation. For additional details on the fitting procedure and results, we refer the interested reader to the Supplemental information.

## Discussion and Conclusions

Process-based modeling facilitates parameterization based on physical considerations and laboratory experiments, and thus helps subsequent application to different real-world scenarios. In this spirit, we proposed a model for bioturbation-induced mixing built upon easily interpretable physical mechanisms, and compared its predictions to laboratory measurements of tracer concentration profiles undergoing redistribution by *L*. *variegatus*. Despite its simplicity, our model proved capable of reproducing a number of key features of the experimental observations.

Our model relies on a burrowing description coupled to a transport rule. The burrowing description assumes independent and memoryless events, and may thus be thought of as a minimal model that captures stochasticity down to the timescale of burrowing events. In a similar fashion, we examined the impact of two different transport rules representing different degrees of tracer mixing during a burrowing event. At early times, model (b) underpredicted shallow-depth concentrations substantially, indicating that the assumption of instantaneous transport of tracer to the maximum depth of each burrowing event underpredicts mixing, especially at short time and depth scales. Another clear feature of model (b) is the prediction of a concentration peak that migrates downward over time while remaining constant in magnitude; if this behavior is observed experimentally, it might indicate that transport is biased towards the bottom of burrowing events. The mean tracer depth was relatively well described in all cases, especially by models (a) and (b). The ADE model significantly overpredicted the profiles’ variance at late times; model (a) provided an adequate description of this metric, but only model (b) captured the flattening-out trend suggested by the experimental data, although it underpredicted the late time values. This suggests that a transport rule with somewhat weaker mixing efficiency than model (a), with tracer redistribution biased towards the bottom of the burrows, may be appropriate to describe our case study. Alternatively, the flattening out of the variance may be an indication of more complex, network-dependent burrowing regimes^22,23^ becoming important at late times, a scenario which is not captured by our assumption of memoryless, independent events. While accounting for variations in burrowing rate or event depth statistics, or considering more complex transport rules, may be easily achieved in the context of a numerical model, it would represent an increase in model complexity and difficulty in parameterization, and we argue that more experimental data is required to warrant a more detailed description. Similarly, we have neglected other processes that may need to be included when modeling other scenarios. Our transport models are simplified descriptions of end member scenarios; sediment deposition above the sediment-water interface is assumed to simply displace the position of the interface, and the description is relative to this moving interface. The bulk sediment physics, in particular processes such as burrow collapse and infill^11^, were not explicitly modeled. While these processes are known to occur, the agreement between model predictions and experimental data suggests that, in the context of our case study, our simplified transport rule (a) was sufficient to adequately capture vertical tracer redistribution.

When compared to a simple advection–dispersion model, our model, in particular with transport rule (a) (full mixing across each burrowing event), generally captured the considered metrics more accurately. Furthermore, the two free parameters *α* and *γ* have clear physical interpretations as the inverse of a mean burrowing depth and a mean burrowing rate. We note that advection–dispersion models with time- or depth-varying advection or dispersion coefficients may yield improved fits. However, it is difficult to physically justify these properties and to parameterize the resulting models, and the depth-dependent exponential dispersion profile we investigated did not lead to improved results. Interestingly, our model also provides a better description than a random walk model parameterized by experimental measurements of waiting time and burrow depth statistics presented in the original study^16^. We interpret this as an indication that the present modeling approach sidesteps the need for a number of assumptions and experimental difficulties involved in obtaining waiting time and burrow depth statistics, thus allowing for easier parameterization.

We emphasize that we have presented a proof-of-concept study for the applicability of minimal process-based models, based on an experimental case-study. As such, further studies of bioturbation by *L*. *variegatus* and other organisms are needed to refine the model and distinguish consistently between burrowing and transport rules. These behaviors are expected to be both coupled and nonstationary in some cases, given that organism activity is linked to external factors such as food availability, temperature, and contaminant concentrations^13,24–26^. We note however that more complex models might not be necessary to capture the features of interest, and may not necessarily yield better results than the simple parsimonious model presented here.

In contrast to more classical models, such as biodiffusion, the present approach provides a more direct and flexible description of mixing by bioturbation, in that it explicitly links mixing dynamics to physical attributes. It also represents a promising basis for parameterizing field observations in terms of major classes of organism behavior (e.g., different modes of feeding and burrowing) in a process-based, transferable manner. This provides a framework where estimates of system-scale biogeochemistry, such as metabolism of sediment organic carbon, are better distinguished from the transport processes upon which they depend^27^.

### Data availability

The dataset analyzed during the current study and the MATLAB source code used for comparing the models to the data are provided online as Supplementary information.

## Electronic supplementary material

Supplementary information

MATLAB code

MATLAB code

MATLAB code

MATLAB code

MATLAB code

Supplementary Dataset 1
