# Supplementary material for: A Process-Based Model for Bioturbation-Induced Mixing
Source: Sci Rep. 2017 Oct 27;7:14287. doi: 10.1038/s41598-017-14705-1 (PMC5660215; doi:10.1038/s41598-017-14705-1)
Supplement: Supplementary file 1 — Supplementary information [file 41598_2017_14705_MOESM1_ESM.pdf]

Supplementary Information for A Process-Based Model for Bioturbation-Induced Mixing

Tomás Aquino[1,2,*], Kevin R. Roche[2,3], Antoine Aubeneau[4], Aaron I. Packman[3], and Diogo Bolster[2]

[1]Spanish National Research Council (IDAEA – CSIC), 08034 Barcelona, Spain
[2]Department of Civil and Environmental Engineering and Earth Sciences, University of Notre Dame, 46556 Indiana, USA
[3]Department of Civil and Environmental Engineering, Northwestern University, 60208 IL, USA
[4]Lyles School of Civil Engineering, Purdue University, 47907 Indiana, USA
[*]tomas.aquino@idaea.csic.es

1 Model fits

Here we offer some details on the fitting metrics discussed in the main text, and show error plots for a range of parameters around the global optimum for each model. We also include results on the exponential dispersion profile model.

The objective function used to compute the model fits is the standard sum of squared errors, summed over all 50 profiles used in fitting. Denoting the data depths (relative to and below the edge of the initial condition) by x_i, the times of each used profile by t_j, the normalized data profile values at each depth and time by c_{ij}, and the corresponding model values by c'_{ij}, it is calculated as:

$$SSE = \sum_{i,j} (c_{ij} - c'_{ij})^2 \ . \tag{S.1}$$

The coefficients of determination r^2 reported in the main text were obtained in the usual way by standardizing the total sum of squared errors in terms of the sum of squared differences of the data points to their mean at each time:

$$\begin{aligned} SSD &= \sum_{i,j} (c_{ij} - \bar{c}_j)^2 \ , \\ r^2 &= 1 - SSE/SSD \ , \end{aligned} \tag{S.2}$$

where the regular arithmetic average computed over all data points at a fixed time is denoted by an overbar.

In Supplementary Fig. S.1 we show the SSE surfaces around the global minimum for each of the models discussed in the main text, including the dispersion model with depth-dependent $D(x) = D_0 e^{-\rho x}$. Note that, as mentioned in the main text, this last model is optimal when $\rho = 0$, in which case it reduces to the regular (advection) dispersion model with $D_0 = D$ and $v = 0$. In Supplementary Fig. S.2 we illustrate how the depth-dependent dispersion model is capable of capturing the late-time profiles better than the regular dispersion model, but fails to simultaneously capture the early-time profiles, leading to an overall worse fit.

2 Data and code files

The data file `Bioturbation_data.mat` provides 242 tracer concentration profiles at specified depths and times, a subset of the experimental data from the original study. It includes the structure `library_data`, which is composed of the following elements:

- `depth_data`: Depth values in mm, relative to the experimentally-determined sediment-water interface.
- `times_data`: Time values in days, relative to the beginning of the experiment.

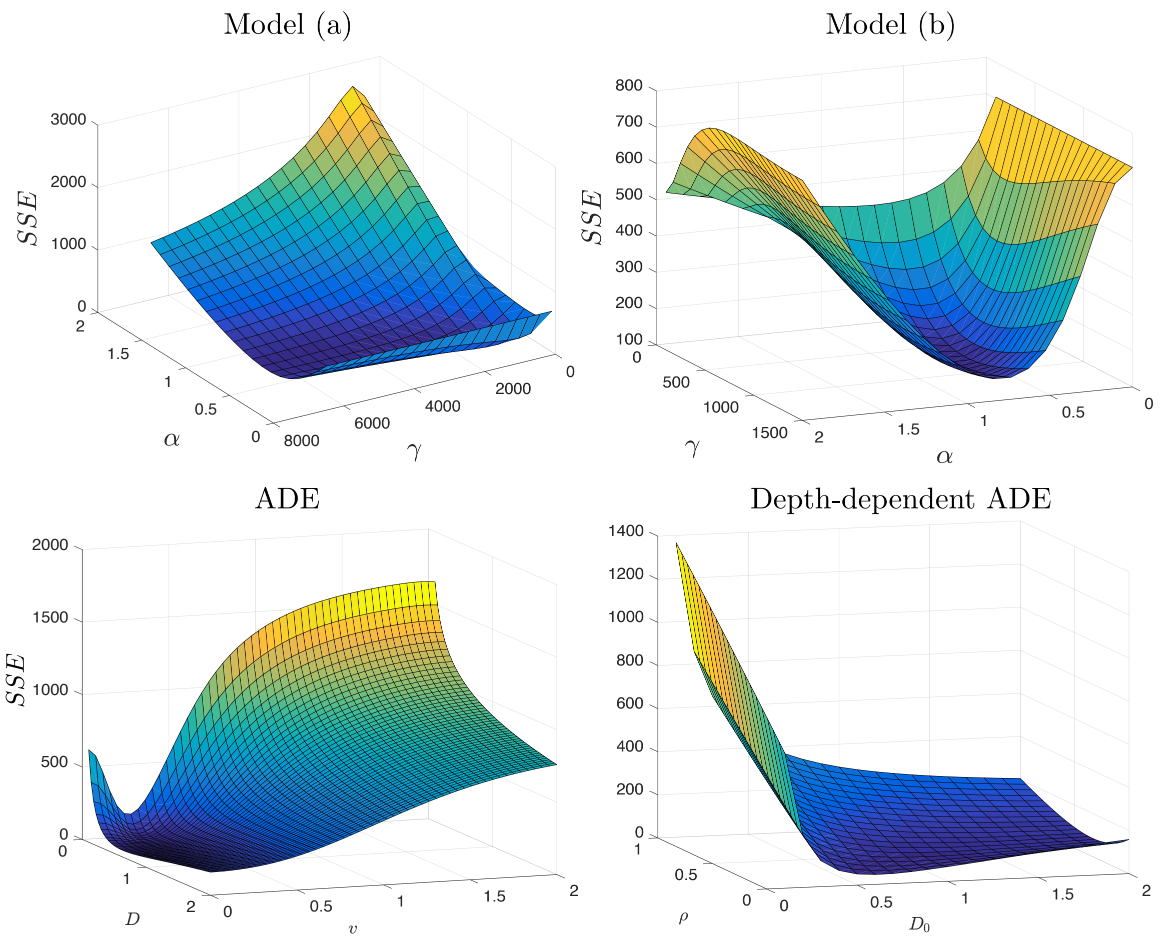

Supplementary Figure S.1: Error surfaces for each model, for a range of parameters around the global minimum.

- `profile_data`: Non-normalized tracer concentration profiles in arbitrary units, at each depth value (lines) and for each time (columns).

The source code files are thoroughly documented through comments. Here we provide a brief description of each file:

- `GetData.m`: Load depths, times, and probability density functions of data profiles for comparison to models.
- `ModelPDF.m`: Compute normalized model concentration profiles.
- `ModelSSE.m`: Compute the total sum of squared errors between theory and data.
- `ModelSSE_plot.m`: Plot the surface of total sum of squared errors between theory and data for given parameter ranges.
- `ModelFit.m`: Compute optimal parameters, total sum of squared errors, and coefficient of determination for model fits.

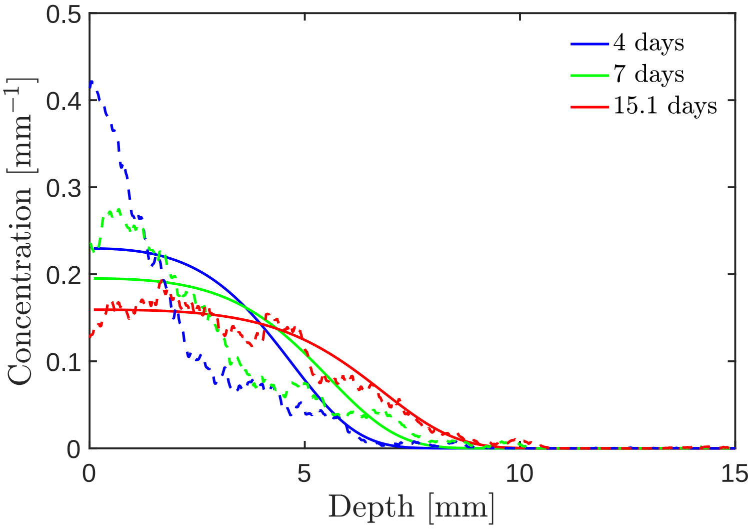

Supplementary Figure S.2: Example fit for the depth-dependent dispersion model, with $D(x) = D_0 e^{-\rho x}$, for $D_0 = 5\,\mathrm{mm}^2\,/\,\mathrm{day}$ and $\rho = 0.5\,\mathrm{mm}^{-1}$, illustrating how this model is capable of capturing the late-time profiles better than the regular dispersion model, but fails to simultaneously capture the early-time profiles, leading to an overall worse fit.
